# Well-being and emotional labor for preschool teachers: The mediation of career commitment and the moderation of social support

**DOI:** 10.1371/journal.pone.0318027

**Published:** 2025-04-24

**Authors:** Xiaojie Su, Delin Yu, Yueyang Yu, Rong Lian

**Affiliations:** 1 Normal School, Urumqi Vocational University, Urumqi, Xinjiang, China; 2 School of Psychology, Fujian Normal University, Fuzhou, Fujian, China; 3 School of Psychology, Nanjing Normal University, Nanjing, Jiangsu, China; Universiti Sains Malaysia - Kampus Kesihatan, MALAYSIA

## Abstract

**Background:**

According to the broaden-and-build theory of positive emotions, well-being is a protective factor against emotional labor. However, it is unclear whether and how well-being contributes to emotional labor for preschool teachers. To investigate the influence of well-being on emotional labor, this study aimed to examine the mediating effect of career commitment between preschool teachers' well-being and emotional labor and whether this effect is moderated by social support.

**Method:**

This study included 363 preschool teachers (*M* = 30.17 years, *SD* = 5.94) in a Chinese province. All participants completed the General Well-Being Schedule, the Emotional Labor Strategy, the Career Commitment Scale, and the Social Support Rating Scale. The data were analyzed using SPSS 26.0.

**Results:**

Correlation analysis revealed a significant positive relationship between well-being, career commitment, social support, and emotional labor. The results of path analysis show that career commitment plays a completely mediating role in the relationship between well-being and emotional labor, which is moderated by social support. The mediating effect of career commitment was significantly greater for individuals with high social support than for those with low social support.

**Conclusion:**

Well-being can promote the emotional labor of preschool teachers by bolstering career commitment, while this process is influenced by social support.

## Background

Emotional labor (EL) refers to individuals adapting their facial expressions and body language to produce emotionally expressive behaviors that meet organizational requirements [1]. The studies on emotional labor were initially focused on sociology, organizational behavior, and management. However, it is gradually gaining attention from researchers in the field of education [2]. Yin and Lee [3] argued that teachers need to regulate their internal feelings and external expressions in their work to show appropriate emotions in their interactions. Teachers need to use a variety of emotional labor strategies based on the rules of emotional expressions requested by students, parents, colleagues, and leaders or perceived by themselves [4]. Thus, teachers are not only physical and mental workers but also highly emotional workers [5]. Due to the unique nature of preschool teachers, they are required to monitor their own emotional performance and to engage in long-term, high-level emotional labor [6]. Research has shown that preschool teachers' emotional labor not only lasts longer than that of teachers working in other grades but also has a much greater intensity of emotional labor, encompassing a wider range of diverse emotional states and interactional targets [7]. In terms of job content, preschool teachers, including observers, good listeners, responsive caregivers, and parenting specialists, are required to play multiple roles [8]. Additionally, preschool teachers are influential for young children. They often serve as a model for young children to learn emotion regulation, helping them to accurately recognize and appropriately express their emotions [9]. Previous research has shown that preschool teachers' emotional labor can promote children's psychological development, foster positive teacher-child relationships, and enhance educational quality [9]. Thus, in recent years, there has been growing concern about how to enhance the emotional labor of preschool teachers. However, the influencing factors and potential mechanisms for emotional labor remain to be clarified.

### The association between well-being and emotional labor

Several previous studies have shown that well-being is intimately linked with emotional labor [10,11]. For example, previous research has demonstrated that well-being is impacted by the use of various emotional labor strategies (i.e., surface acting, deep acting, and the expression of naturally felt emotions) among teachers [12]. Most research suggests that deep acting and the expression of naturally felt emotions positively predict well-being [13]. From the perspective of emphasizing positive emotions, surface acting is not conducive to well-being [14,15]. However, from the perspective of emotional value, surface acting is not all “bad” for employees [16]. In a 3-week study of 79 managers, pretending to be positive was found to be unhelpful for well-being, but pretending to be negative increased well-being [16]. In addition, a meta-analysis indicated a strong relationship between surface acting and indicators of impaired well-being, while deep acting had a weak relationship with these indicators [17]. These inconsistent results may imply that the association between emotional labor and well-being is intricate.

According to the broaden-and-build theory of positive emotions [18], positive emotions broaden an individual's accessible repertoire of thoughts and action urges, helping individuals increase their cognitive flexibility, expand their attention span, and provide them with long-term adaptive benefits [19]. Previous research has suggested that positive psychological capital can facilitate job performance, including emotional labor. For example, it has been revealed that positive psychological capital promotes emotional labor for corporate employees and managers [20], and this finding was revalidated in a later study of employees in the airline service industry [21]. In addition, Tosten and Toprak [22], focusing on teachers, found that positive psychological capital positively predicts the use of emotional labor behaviors. Well-being is part of positive psychological capital [23], which may also positively predict emotional labor. Therefore, we propose H1: Well-being is positively associated with emotional labor for preschool teachers.

### Career commitment as a mediator

According to Blau [24], career commitment is an attitudinal measurement of individual motivation to work in a chosen career, which is a psychological link between a person and his or her career based on an affective reaction to the career [25]. Individuals with higher levels of career commitment tend to be more willing to take on tasks, exhibit exceptional work performance, and experience more positive emotional states at work [26]. Therefore, preschool teachers who possess greater career commitment are more frequently engaged in proactive behaviors aimed at achieving their objectives [27]. Previous research has shown that well-being positively predicts career commitment for teachers [28]. Consistent results have also been observed in preschool teachers. For example, Wang [29] reported that career well-being promotes preschool teachers' career commitment.

The correlation between emotional labor and career commitment has been consistently shown in the literature [30]. Chang [31] indicated that when employees' career expectations are met, their motivation and work incentives increase. Huynh et al. [32] reported that career commitment predicted emotional labor in caregivers. Furthermore, there was a significant positive correlation between teachers' emotional labor and career commitment [33]. Based on the results of Blau’s career commitment questionnaire, it appears that his conception of career commitment is centered around career emotions, such as loving and wanting to continue in his current work [34]. Therefore, we propose H2: Career commitment mediates the association between well-being and emotional labor.

### Social support as a moderator

Social support can be divided into three segments: objective support, subjective support, and the individual's utilization of social support [35–37]. Social support can be defined as the subjective and objective assistance that individuals receive from their social network, as well as their own utilization of such support [37]. Social exchange theory [38,39] suggests that employees seek a balance between their orientation toward the organization and the organization's orientation toward them through norms of reciprocity [40], and employees receiving organizational support enhance their motivation to contribute to organizational goals [41]. In addition, organizational support theory suggests that perceived organizational support results in a sense of obligation to achieve organizational goals, which promotes organizational commitment [42]. Therefore, when employees perceive support from their organization, they feel obligated to care about the organization and contribute to its goals [43]. According to previous theories, the association between preschool teachers' career commitment and emotional labor may depend on perceived social support, although this view has not yet been confirmed.

Various studies have revealed a correlation between increased social support and improved individual performance [44–46]. Perceived social support is an important mechanism for promoting career commitment [47]. A meta-analysis of organizational support suggested that organizational support is positively related to commitment and can improve employee motivation, satisfaction, and well-being; reduce work stress; and thus buffer or regulate employees' burnout caused by emotional labor [48]. Recent research suggests that greater career commitment and enthusiasm emerge among preschool teachers who receive more social support [49]. Employees who receive social support tend to engage in behaviors that are beneficial to the organization and develop positive work attitudes [43]. Research shows that the impact of preschool teachers' career commitment to emotional labor can be facilitated by social support. Therefore, we propose H3: The mediating effect of career commitment on the association between well-being and emotional labor is moderated by social support.

### The present study

Previous theoretical and empirical research suggests that well-being has a significant association with emotional labor. However, the mediating and/or moderating mechanisms underlying this association are still unclear. In the present study, based on the broaden-and-build theory of positive emotions, psychological capital theory, and social exchange theory, we construct a moderated mediation model. The present study examines the effect of well-being on emotional labor and the mediating role of career commitment for preschool teachers, as well as whether this process is moderated by social support. A complementary investigation of the impact of psychological capital is provided by demonstrating the mediating role of career commitment between the well-being and emotional labor of preschool teachers. It examines how social support moderates the association between career commitment and emotional labor in relation to the reciprocity norm of social exchange theory. Through this study, new empirical support can be provided for social exchange theory, which in turn will enrich the theoretical aspects of occupational psychology and organizational behavior.

The significance of this study lies not only in its contribution to theory but also in its guidance for practical application. The study will be able to provide targeted educational recommendations for the education sector and offer a scientific basis for relevant policy making. Preschool teachers are able to work in a more motivational and supportive work environment, which promotes their professional growth and enhances their well-being, thereby contributing to the continued development and progress of the entire education sector.

## Methods

### Participants

The participants in the study were preschool teachers with tenure at public kindergartens from a province of China. A total of 425 questionnaires were sent out, and 401 were returned, for a return rate of 94.35%. Invalid questionnaires were excluded according to the regularity of the responses (e.g., almost every item had the same answer), and 363 valid questionnaires were returned, for a validity rate of 90.52%. The mean age of the participants was 30.17 years (*SD* = 5.94), and the mean length of teaching was 5.24 years (*SD* = 4.94). A total of 335 females and 28 males were included in the final sample.

### Measurements

#### General Well-Being Schedule (GWBS).

This study used the General Well-Being Schedule developed by Fazio [50] and revised by Duan [51]. The 18-item scale consists of six dimensions: satisfying interesting life (2 items), health worry (2 items), energy level (4 items), emotional-behavioral control (3 items), depressed-cheerful mood (3 items), and relaxed versus tense-anxious (4 items) [52]. Scoring utilized 5-point (items 2, 5, 6, 7), 6-point (items 1, 3, 4, 8–14), and an 11-point scale was used for items 15–18. Higher scores indicate higher levels of well-being. In the present study, the Cronbach’s α for this scale was 0.813, indicating good internal consistency.

#### Emotional Labor Strategy (ELS).

The Emotional Labor Strategy for preschool teachers revised by Lin [53] was used in the present study. This questionnaire consists of 10 items to which the participants respond on a 5-point Likert scale from 1 (not at all true) to 5 (very true). It comprises three dimensions: surface acting (3 items), deep acting (4 items), and the expression of naturally felt emotions (3 items). In the present study, the Cronbach’s α for the scale was 0.735, indicating acceptable internal consistency.

#### Career Commitment Scale.

The present study used the Career Commitment Scale designed by Blau [34], which consists of 7 items on a 5-point Likert scale ranging from 1 (strongly disagree) to 5 (strongly agree), with higher scores indicating higher levels of career commitment. In the present study, the Cronbach’s α for this scale was 0.933, indicating high internal consistency.

#### Social Support Rating Scale (SSRS).

This study employed the Social Support Rating Scale created by Xiao [37], which comprises 10 items, including subjective support (4 items), objective support (3 items), and support utilization (3 items). In the present study, the Cronbach’s α for this scale was 0.775, indicating acceptable internal consistency.

### Procedures

The study was conducted after a training project for preschool teachers; a PhD in psychology pronounced the instructions on the spot and requested that the participants complete the questionnaire within a specified time. The questionnaire was collected via the SoJump platform (https://www.wjx.cn/). After receiving information about the study, participants provided written informed consent. The participants were directed to the next screen to complete the questionnaire if they agreed to take part in the study. The study was approved by the Ethics Committee of the School of Psychology, Fujian Normal University (PSY230050), and the participants were recruited for this study between 5 December 2023 and 16 January 2024.

All the statistical analyses were conducted in SPSS 26.0. Descriptive statistical analysis and Pearson correlation analysis were used to test the variables. Then, the simple mediating effect of career commitment on the association between well-being and emotional labor was tested using Model 4 of PROCESS 3.3. Finally, the moderated mediation effect was tested using Model 14 of PROCESS 3.3.

## Results

### Common method bias

The Harman single-factor test was used to test for common method bias and estimate common method bias across all items [54]. The results showed that the eigenvalues of 13 factors were greater than 1. The first factor explained 19.52% of the variance (below the critical threshold of 40%), which was less than the critical standard of 40%, indicating that no serious common method bias was present in the data.

### Correlation analysis between variables

Descriptive statistical analysis for emotional labor, career commitment, social support, and well-being and correlations between these variables are presented in Table 1. We found that emotional labor was positively correlated with career commitment (*r* = 0.495, *p* < 0.001), social support (*r* = 0.211, *p* < 0.001), and well-being (*r* = 0.182, *p* < 0.001). Career commitment was positively correlated with social support (*r* = 0.374, *p* < 0.001) and well-being (*r* = 0.376, *p* < 0.001). The results of the analyses also revealed that social support was positively correlated with well-being (*r* = 0.342, *p* < 0.001). Based on the present results, H1 was **confirmed**, and the close association between the variables in this study fits the requirements of the moderation effect analysis and is suitable for further mediation and moderation analysis.

**Table1:** Descriptive statistics and correlation coefficients for all variables.

Variables	*Mean*	*SD*	1	2	3	4
1 Emotional Labor	38.10	5.67	1			
2 Career Commitment	24.04	6.59	0.495^***^	1		
3 Social Support	38.93	7.50	0.211^***^	0.374^***^	1	
4 Well-being	74.67	13.82	0.182^***^	0.376^***^	0.342^***^	1

*Note.* ^***^ *p* < 0.001.

### Testing for mediation effect

The mediating effect of career commitment was tested using Model 4 of the PROCESS developed by Hayes [55]. When the mediating variable career commitment was added (Table 2), the positive predictive effect of well-being on career commitment was significant (*β* = 0.376, *t* = 7.720, *p* < 0.001), and the positive predictive effect of career commitment on emotional labor was significant (*β* = 0.497, *t* = 10.057, *p* < 0.001); however, the predictive effect of well-being on emotional labor was insignificant (*β* = -0.005, *t* = -0.100, *p* = 0.921). In addition, the 95% CI for the mediating effect of career commitment on well-being and emotional labor showed that neither the low-level group nor the high-level group had a coefficient of 0 (Table 3), confirming the significant indirect effect of career commitment on the association between well-being and emotional labor. However, the direct effect was not significant. Therefore, career commitment fully mediates the effect of well-being on emotional labor, confirming H2.

**Table 2:** Testing the mediation effect of career commitment.

Regression equation（*N*=363）		Overall fit index			Coefficient Significance	
Result Variables	Predictive variables	*R*	*R²*	*F*	*β*	*t*
Career Commitment	Well-being	0.376	0.142	59.598^***^	0.376	7.720^***^
Emotional Labor	Well-being	0.495	0.245	58.483^***^	-0.005	-0.100
	Career Commitment				0.497	10.057^***^

*Note.* Variables entered into the regression equation were standardized. The same below.

*** *p* < 0.001

**Table 3:** Bootstrapping estimates of 95% confidence intervals of (CI) estimation for the mediating effect.

Routes	*Effect Value*	*Boot SE*	*Boot CI* *Lower limit*	*Boot CI* *Upper limit*	*Relative Effect Value*
Total effect	0.182	0.052	0.080	0.284	
Direct effect	-0.005	0.049	-0.102	0.092	-2.69%
Mediating Effect	0.187	0.035	0.124	0.259	102.69%

### Testing for moderation effect

**Fig 1:**
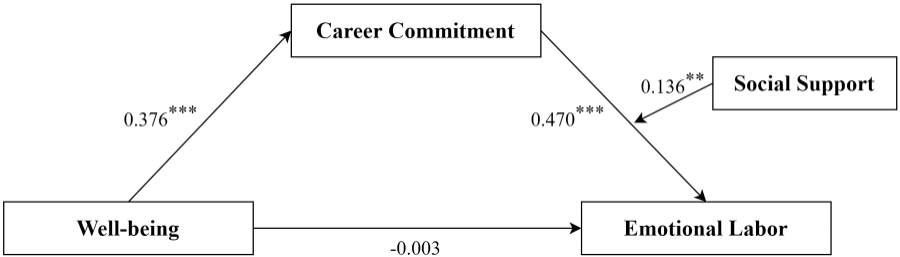
The moderated mediation model.

The moderated mediating effect (Fig 1) was tested using Model 14 of the PROCESS developed by Hayes [55]. The mediating effect of career commitment was significant (*effect* = 0.187, *p* < 0.05). Moreover, (Table 4) the interaction effect between career commitment and social support on emotional labor was significant (*β* = 0.136, *t* = 2.938, *p* < 0.01), indica*t*ing that social support moderated the predictive effect of career commitment on emotional labor. Besides, the 95% CI for the moderated mediation effect showed that neither the low-level group nor the high-level group contained 0. Thus, a moderating effect of social support was confirmed.

**Table 4:** Testing the moderated mediation effect of well-being on emotional labor.

Regression equation (*N*=363)		Overall fit index			Coefficient Significance	
Result Variables	Predictive variables	*R*	*R²*	*F*	*β*	*t*
Career Commitment	Well-being	0.376	0.142	59.598^***^	0.376	7.720^***^
Emotional Labor	Well-being	0.514	0.264	32.077^***^	-0.003	-0.060
	Career Commitment				0.470	9.157^***^
	Social Support				0.039	0.768
	Career Commitment × Social Support				0.136	2.938^**^

*Note.* ^**^ *p* < 0.01, ^***^ *p* < 0.001.

**Fig 2:**
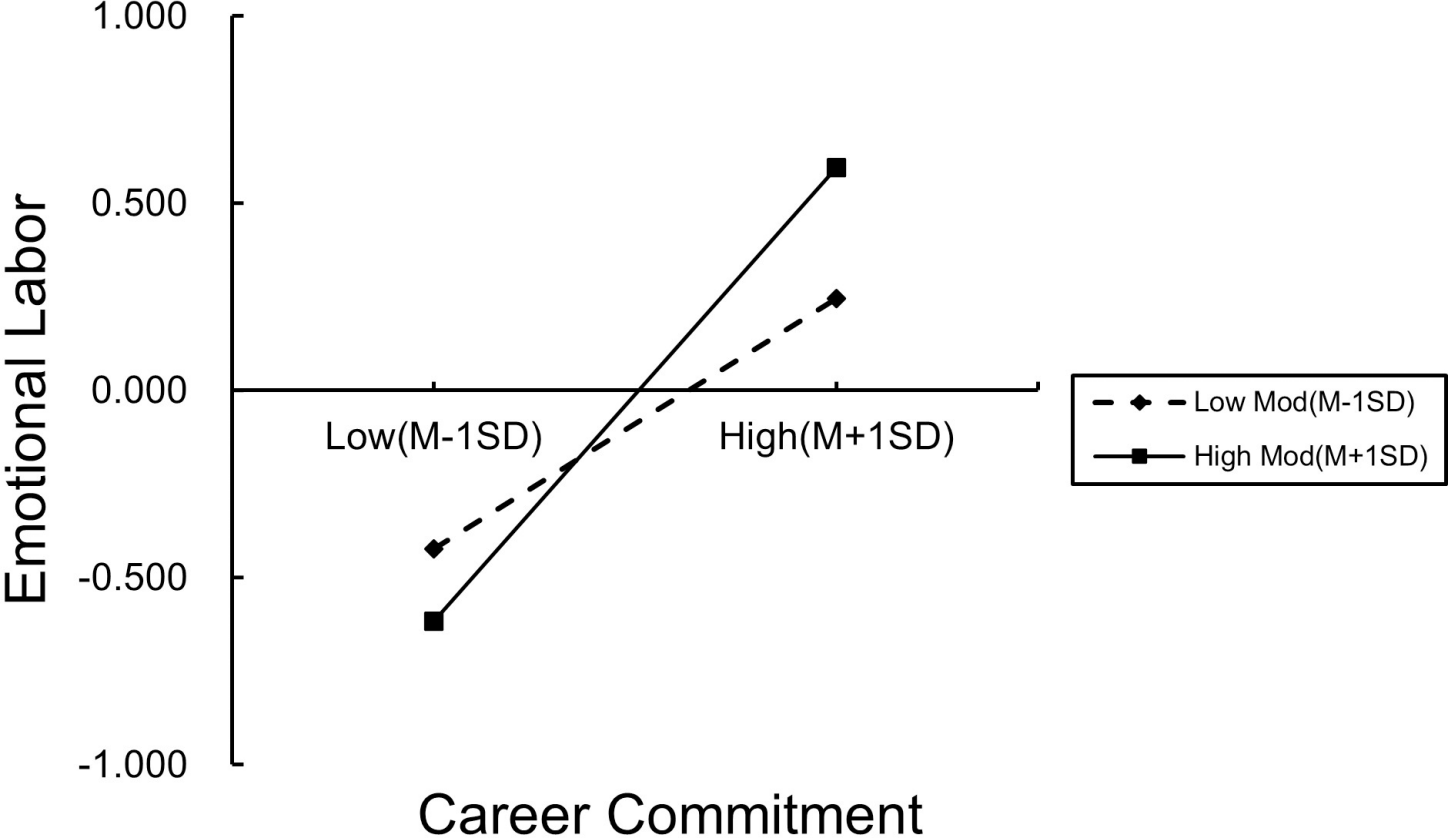
The moderating role of social support in career commitment and emotional labor. The solid line on the left represents the value of emotional labor under high social support and low career commitment, and the solid line on the right represents the value of emotional labor under high social support and high career commitment; the dotted line on the left displays the value of emotional labor under low career commitment and low social support; and the solid line on the right is the value of emotional labor under low social support and high career commitment.

Furthermore, a simple slope analysis was used to demonstrate the interaction effect at low (*M -* 1*SD*) and high (*M +* 1*SD*) levels of social support (Table 5). Regardless of whether social support was at a low level or a high level, the 95% CI for the predictive effect of career commitment on emotional labor showed that neither the low-level group nor the high-level group received 0. Compared with low social support, career commitment had a stronger effect on emotional labor among preschool teachers with high social support (Fig 2). That is, high social support was able to enhance the predictive effects of career commitment on emotional labor.

**Table 5:** Mediating effects at different levels of social support.

Routes	*Effect value*	*Boot SE*	95% Confidence interval	
			*Boot CI Lower limit*	*Boot CI Upper limit*
Intermediary Effect (*M -* 1*SD*)	0.126	0.033	0.067	0.196
Intermediary Effect M	0.177	0.034	0.118	0.249
Intermediary Effect (*M +* 1*SD*)	0.228	0.044	0.150	0.322
Amount of adjustment for mediating effects	0.051	0.019	0.016	0.091

The Johnson–Neyman technique was used to identify the regions in the range of the moderator variable where the effect of career commitment on emotional labor is statistically significant [56,57]. The results indicate that for the substitute value of social support (after standardization) in the interval [-1.913, 2.274], career commitment has a significant effect on emotional labor, which means that the effect of career commitment on emotional labor increases with increasing social support. The effect of career commitment on emotional labor is nonexistent when social support is low.

Therefore, H3 proved that the mediating effect of career commitment on the association between well-being and emotional labor is moderated by social support.

## Discussion

The moderated mediation model was used to examine the effect of well-being on emotional labor and the underlying mechanisms involved. For preschool teachers, career commitment was a mediator between well-being and emotional labor, and social support had a moderating effect on the latter half of the path of the mediating effect. Overall, the results support the research hypothesis.

### The association between preschool teachers’ well-being and emotional labor

Our findings provide compelling evidence for the intricate relationship between well-being and emotional labor among preschool teachers, with career commitment playing a pivotal mediating role. We found that well-being was significantly related to emotional labor, which is consistent with previous conclusions [10,11]. Prior research focusing on the effects of emotional labor on well-being has shown that deep acting and the expression of naturally felt emotions positively predict well-being [58], while surface acting negatively predicts well-being [59,60]. It has also been shown that if negative emotions on surface acting are fake, the potential emotions will be positive [16]. There is also a positive relationship between surface acting and well-being [61]. Therefore, the relationship between emotional labor and well-being is complex [62]. According to the broaden-and-build theory of positive emotions [18], positive emotions can consistently enhance individual resilience and work performance, suggesting that well-being can also promote emotional labor, which was supported by the present study.

However, our study extends beyond simply establishing this association by elucidating the underlying mechanism through which well-being influences emotional labor. We found that well-being significantly and positively predicts career commitment, which is consistent with psychological capital theory. Well-being is an essential component of psychological capital [63]. From the perspective of this theory, psychological capital can facilitate the integration of various internal and interpersonal resources [64], and this integration effectively increases workers' level of career commitment. Previous empirical research has also confirmed that psychological capital is significantly and positively related to employees' career commitment [65,66].

The results showed that career commitment significantly predicted emotional labor positively and mediated the association between well-being and emotional labor, which is consistent with the hypothesis of this study. Teacher career commitment is essential to the professional development of teachers and is directly related to their work engagement and teaching quality [26]. Previous empirical research has also confirmed that teachers' career commitment is a positive affective attachment [67,68] that facilitates emotional regulation and adaptation at work, thereby promoting emotional labor [69]. Buettner et al. [70] also reported that positive emotional states promote career commitment and that career commitment contributes to emotional labor [71]. Therefore, career commitment mediates the relationship between well-being and emotional labor.

Consistent with the hypothesis of this study, our results indicated that well-being does not directly affect emotional labor but only indirectly promotes emotional labor by increasing career commitment. The mediating variable of career commitment, which plays an essential role in the process of well-being facilitating emotional labor, has a full mediating effect. The results revealed that career commitment may facilitate the process of transforming preschool teachers' well-being toward emotional labor. Career commitment is a psychological link between a person and his or her career and is based on an affective reaction [25]. An increase in career commitment may promote teachers' adaptation and attachment to the organization and increase their affective connection, which in turn may contribute to the easier conversion of well-being to emotional labor.

### Moderating effect of social support

The current study also revealed that the mediating effect of career commitment on well-being and emotional labor was moderated by social support. Career commitment, affected by well-being, promotes emotional labor when preschool teachers receive adequate social support, and this effect disappears when social support is inadequate [72].

The results are consistent with those of social exchange theory and the reciprocity norm hypothesis [42,73]. Furthermore, according to the conservation of resources model theory [74], social support is a protective factor that promotes the expression of positive emotions [75]. From this perspective, the negative effects of emotional labor on preschool teachers may be weakened by social support. Hence, preschool teachers are better equipped to fulfill their career commitments related to emotional labor when they receive substantial social support. This view is also supported by previous research [43]. The care and assistance offered by social support contributed to preschool teachers' more positive attitudes, more harmonious work atmosphere, and more ample emotional interactions [76]. Theoretical and empirical findings suggest that social support is an important guarantee for career commitment to promote emotional labor in preschool teachers.

### Research Implications

The results theoretically support the moderated mediation model mechanism between well-being and emotional labor. The present study is the first to demonstrate the mediating role of career commitment between well-being and emotional labor. Then, the conditions under which the mediating function existed, that is, the latter portion of the mediating path, were moderated by social support. Overall, our understanding of the intrinsic association between variables and the interaction between preschool teachers' well-being and career development is enhanced.

Based on the moderated mediation model, relevant education authorities and schools should recognize that the greater the perceived well-being of teachers is, the more likely they are to provide emotional labor more effectively as a result of the influences of their career commitment and the social support they receive. Improvements can be made to the current situation in three directions. From the legal and regulatory perspective, teachers' unions, as administrative entities, must actively uphold existing legislation, specifically the Teachers Law of the People’s Republic of China and the Education Law of the People’s Republic of China, ensuring that teachers' salaries, social security, and other benefits are fully implemented. Such measures are crucial for enhancing the subjective well-being of preschool teachers. Secondly, the establishment of a designated administrative body for preschool teachers is recommended, with the implementation of a tiered professional development program incorporating diversified training and support initiatives [77]. These should be aimed at enhancing teachers' career commitment and emotional labor skills. Finally, it is essential that legal frameworks are in place to guarantee that teachers receive adequate social support, with the responsibilities of the government, educational institutions, and society as a whole clearly defined. From the school management perspective, multifaceted support should be provided by schools, including providing kind and humane treatment, creating an organizational atmosphere of solidarity and cooperation, and implementing a fair system of rewards and punishments. A positive organizational environment fosters emotional connections among preschool teachers, increases their well-being and positive emotional experiences, and enhances their career commitment, thus yielding favorable effects conducive to emotional labor. From the individual capacity perspective, activities such as workshops, cognitive reappraisal consultation groups, and theme salons can develop teachers' emotional management skills [78] and help them to accurately perceive, understand, and effectively regulate their emotions. All of the above factors help preschool teachers maintain a high level of professional well-being and make meaningful contributions to the field of preschool education through the delivery of qualitative emotional labor.

### Limitations

The present study has the following limitations. First, the investigation was cross-sectional, so the causal relationship between the variables should be further tested by employing longitudinal research. Second, the sample size of this study was relatively small, and the use of teachers from specific preschools in the Western region of China to represent the preschool teacher population may introduce bias, which may constrain the external validity of the findings. Future research should expand the sample size to include preschool teachers from different countries, ages, and cultural backgrounds to explore the generalizability of the association between preschool teachers' well-being and emotional labor. Third, we acknowledge that conducting this study after the completion of a training program for preschool teachers may have influenced the sample characteristics and study outcomes. Specifically, the training program may have enhanced teachers' professional awareness, emotional labor skills, or social support, which could have affected their responses to the questionnaire. Therefore, when interpreting these results, we must consider the potential influence of recent training experiences on participants, which may limit the generalizability of our findings. Future research should employ a longitudinal research design to assess teachers at various stages of the training program (e.g., before training, immediately after training, and some time after training) to gain a more comprehensive understanding of the effects of the training program on teachers' various aspects. Finally, the subjective reporting method was adopted to measure well-being, career commitment, social support, and emotional labor in this study, and there may have been a socially desirable response to the results. Therefore, a combination of methods, such as peer evaluation or behavioral experiments, should be used to test the current conclusions.

## Conclusion

In summary, the present study tested the association between well-being and emotional labor in preschool teachers. Well-being is positively associated with emotional labor. Career commitment fully mediates the association between well-being and emotional labor, and this mediating effect is moderated by social support. Despite some limitations, the present study contributes to a better understanding of the association between well-being and emotional labor, and the results emphasize the important role of career commitment and social support. This study also provides practical guidance for enhancing preschool teachers' emotional labor.

## Supporting information

S1 File Appendix independent samples t-Tests removed vs retained samples. (ZIP)
